# Betulin - a plant-derived cytostatic drug - enhances antitumor immune response

**DOI:** 10.1186/2051-1426-2-S3-P175

**Published:** 2014-11-06

**Authors:** Kathrin Pfarr, Corina Danciu, Cristina Dehelean, Josef M Pfeilschifter, Heinfried H Radeke

**Affiliations:** 1 Immune Pharmacology, pharmazentrum, Clinic of the Goethe University, Frankfurt/Main, Germany; 2 University of Medicine and Pharmacy „Victor Babes", Timisoara, Romania

## 

Conventional cytostatic cancer treatments are rarely curative. New therapeutic strategies are more promising by targeting the tumor microenvironment, inhibiting angiogenesis and antagonizing the immunosuppressive activity of established tumors. Along these lines, plants provide a broad spectrum of potential drugs for cancer therapy, such as betulin, a pentacyclic triterpene of the birch tree representing the reduced congener of betulinic acid. An anticancer activity of betulinic acid has been shown already and was linked to its ability to directly trigger mitochondrial membrane permeabilization, a central event in the apoptotic process. In contrast to the potent cytostatic action towards different tumor cell lines, non-neoplastic cells and normal tissue remain relatively resistant.

This study aimed to investigate the antitumor activity on melanoma cells and the immune modulating effect on dendritic cells and T cells of the phytocompound betulin to improve the melanoma immunotherapy by this adjuvant. By means of diverse proliferation assays as well as nuclear DAPI staining we could confirm that betulin decreased the proliferation rate of both the highly metastatic B16F10 and the low metastatic B164A5 melanoma cell lines. In addition, annexin V/7-aminoactinomycin D staining revealed that betulin induced a higher rate of apoptosis in melanoma cells as compared to primary bone marrow derived dendritic cells (BMDCs) of C57BL/6 mice. Furthermore, as evaluated by co-incubation assays and ELISA we found that betulin significantly stimulated the Toll-like-receptor-4-dependent IL-12p70 production of murine BMDCs (n = 3, p ≤ 0.01). We could further show that this increased secretion of IL-12p70 protein was due to an increased IL-12p35 mRNA expression, while the mRNA level of the IL-12p40 subunit remained unchanged (n = 10, p ≤ 0.001). Interestingly, other cytokines like IL-6, IL-10 or IL-23 were also not affected by treatment with betulin. Subsequent *ex vivo *experiments utilizing transgenic spleen cells revealed that the betulin-enhanced activation of dendritic cells is transmitted to an increased, antigen-specific T cell stimulation as demonstrated by an induction of IL-2 and interferon-gamma in ELISA and ELISPOT analysis (n = 3, p ≤ 0.5). A next step would be to load dendritic cells with specific melanoma antigens to receive a directed immune response against the tumor tissue and confirm these results *in vivo*.

In summary, cytostatic agents like betulin that simultaneously exhibit immune stimulatory activity hold great promise as a novel approach for an integrated cancer therapy.

